# Development of High Dielectric Electrostrictive PVDF Terpolymer Blends for Enhanced Electromechanical Properties

**DOI:** 10.3390/nano11010006

**Published:** 2020-12-22

**Authors:** Il Jin Kim, Kie Yong Cho, Eunji Kim, Young Je Kwon, Min Young Shon, Bo-In Park, Seunggun Yu, Jin Hong Lee

**Affiliations:** 1 School of Chemical Engineering, Pusan National University, Busan 46421, Korea; ijkim@kiflt.re.kr (I.J.K.); eunjikim@pusan.ac.kr (E.K.); 2 Department of Industrial Chemistry, Pukyong National University, 45 Yongso-ro, Nam-gu, Busan 48513, Korea; kieyongh@pknu.ac.kr (K.Y.C.); myshon@pknu.ac.kr (M.Y.S.); 3 Division of Applied Chemical Engineering, Pukyong National University, 45 Yongso-ro, Nam-gu, Busan 48513, Korea; a01072524246@gmail.com; 4 Department of Materials Science and Engineering, Korea Advanced Institute of Science and Technology, Daejeon 34141, Korea; boin0905@gmail.com; 5 Insulation Materials Research Center, Korea Electrotechnology Research Institute (KERI), Changwon 51543, Korea

**Keywords:** PVDF, polymer composites, actuators, electromechanical properties, high dielectric constant

## Abstract

Electroactive polymers with high dielectric constants and low moduli can offer fast responses and large electromechanical strain under a relatively low electric field with regard to theoretical driving forces of electrostriction and electrostatic force. However, the conventional electroactive polymers, including silicone rubbers and acrylic polymers, have shown low dielectric constants (ca. < 4) because of their intrinsic limitation, although they have lower moduli (ca. < 1 MPa) than inorganics. To this end, we proposed the high dielectric PVDF terpolymer blends (PVTC-PTM) including poly(vinylidene fluoride-trifluoroethylene-chlorofluoro-ethylene) (P(VDF-TrFE-CFE), PVTC) as a matrix and micelle structured poly(3-hexylthiophene)-*b*-poly(methyl methacrylate) (P3HT-*b*-PMMA, PTM) as a conducting filler. The dielectric constant of PVTC-PTM dramatically increased up to 116.8 at 100 Hz despite adding only 2 wt% of the polymer-type filler (PTM). The compatibility and crystalline properties of the PVTC-PTM blends were examined by microscopic, thermal, and X-ray studies. The PVTC-PTM showed more compatible blends than those of the P3HT homopolymer filler (PT) and led to higher crystallinity and smaller crystal grain size relative to those of neat PVTC and PVTC with the PT filler (PVTC-PT). Those by the PVTC-PTM blends can beneficially affect the high-performance electromechanical properties compared to those by the neat PVTC and the PVTC-PT blend. The electromechanical strain of the PVTC-PTM with 2 wt% PTM (PVTC-PTM2) showed ca. 2-fold enhancement (0.44% transverse strain at 30 V_pp_ μm^−1^) relative to that of PVTC. We found that the more significant electromechanical performance of the PVTC-PTM blend than the PVTC was predominantly due to the electrostrictive force rather than electrostatic force. We believe that the acquired PVTC-PTM blends are great candidates to achieve the high-performance electromechanical strain and take all benefits derived from the all-organic system, including high electrical breakdown strength, processibility, dielectrics, and large strain, which are largely different from the organic–inorganic hybrid nanocomposite systems.

## 1. Introduction

Electroactive polymers (EAPs) have been investigated for potential applications, such as electrical sensors, compact soft actuators, artificial muscles, and micro-robotics [1,2,3,4,5,6,7]. That is because of the several attractive advantages of EAPs, including sizeable electromechanical strain, high power-to-mass ratio, fast response, lightweight, and low cost [1,2,3,4,5,6,7]. When an electric field is applied across a film, electrostrictive materials are conventionally squeezed in the thickness direction and stretched in the transverse direction. This result came from Maxwell stress originating from the Coulomb interaction between oppositely charged compliant electrodes [1,2,3]. The resulting electromechanical actuation strain is expressed by Equation (1) as follows:$\;S=\frac{-\varepsilon_{r}\varepsilon_{0}E^{2}}{2Y}$ where *ε_0_* is permittivity at free space (8.82 × 10^−12^ F m^−1^) and *E* is applied electric field. *Y* and *ε_r_* are modulus and dielectric constant, respectively [6,8,9]. This equation reveals that the EAP materials generally require a high dielectric constant and low modulus for high-performance electromechanical strain.

To date, various EAPs have been developed, such as a dielectric elastomer, an electrostrictive graft elastomer, electro-viscoelastic elastomers, a liquid crystal elastomer, and ferroelectric polymers [5,10,11]. PVDF and its copolymers are well-known materials for strong piezoelectricity. In particular, PVDF and P(VDF-TrFE) have been used for the fabrication of electromechanical devices owing to their efficient electrical-to-mechanical energy conversion [12]. However, those still have limited dielectric constants, large hysteresis because of large permanent polarization domains, and the formation of long-range polar ordering [13]. To this end, Zhang et al. have introduced by employing defect modifications, a bulky third monomer CFE. It has been shown that the ferroelectric polymers can be converted into a ferroelectric relaxor material by the post-treatment process with physical and thermal stimulations [14,15]. The ferroelectric relaxor polymers, including poly(vinylidene fluoride-trifluoroethylene-chlorofluoro-ethylene) (P(VDF-TrFE-CFE), PVTC), have been exploited in emerging electrostrictive materials for replacing conventional EAPs. However, ferroelectric relaxor polymers still have suffered from limitations, including the requirement of relatively strong external electric fields for a high-performance electromechanical property.

A high dielectric constant and a low modulus are required to achieve a high electromechanical strain at the low external electric field. Various conducting organic and inorganic fillers have been proposed to increase the dielectric constants of the polymer matrixes [7,11,16,17,18,19,20]. Carbon nanofillers and polyaniline conducting polymers have been proved as great candidates to enhance the electromechanical properties of the polymer composites [21,22]. However, it is difficult to obtain a homogeneous dispersion of the fillers in the polymer matrix because of the undesired attractive force between the fillers and a lack of interfacial interaction between filler and EAPs, which readily lead to the agglomeration state of the fillers. The inhomogeneous dispersions deteriorated the dielectric insulation property, resulting in many side effects, including increased dielectric loss and decreased electrical breakdown strength. In addition, the inorganic conducting fillers have shown detrimental effects in the range of the operational electric field. Due to those considerations, the insulating group grafting approach from the organic conducting fillers has been proposed [23,24,25,26]. Specifically, the strategies for the enclosing of the copper–phthalocyanine and conductive polyaniline fillers by insulating groups based on the chemical grafting method allowed enhanced electromechanical properties in addition to reducing the sacrifice of the dielectric loss and the electrical breakdown strength. That all occurs because the grafted insulating groups can prevent the aggregation of the conducting filler and help the uniform dispersion by inter-chain interaction with the polymer matrix [27,28]. 

In this study, we suggest that PVTC blends including the poly(3-hexylthiophene)-*b*-poly (methyl methacrylate) (P3HT-*b*-PMMA) conducting fillers for all organic blend systems yield PVTC-PTM blends with minimal moduli increases and maximized dielectric constant growth. The PVTC-PTM blends were prepared by a conventional solution casting method. The acquired PVTC-PTM blends were examined for their compatible blend and crystalline properties, which are important parameters to determine the electromechanical properties of electroactive polymers. The electromechanical properties of the PVTC-PTM blends were evaluated by measuring their transverse strain under the electric field. They were compared with prepared reference materials, including neat PVTC and PVTC blends with a P3HT homopolymer (PVTC-PT), along with reported electroactive polymers, including silicone rubbers and acrylic polymers. The electromechanical strain mechanisms of the PVTC-PTM were systematically addressed in this study based on the parameter (dielectric constant and modulus) effects and crystalline phase transition behavior.

## 2. Materials and Methods 

### 2.1. Materials

All chemicals were purchased from Sigma-Aldrich (St. Louis, MO, USA) and used without purification process unless mentioned otherwise. P3HT-*b*-PMMA block copolymer was prepared by using the reported synthetic method [29,30,31,32]. P(VDF-TrFE-CFE) (61/29/10 mol%) was synthesized using the reported synthetic route [13].

### 2.2. Preparation of P(VDF-TrFE-CFE)/P3HT-b-PMMA (PVTC-PTM) and P(VDF-TrFE-CFE)/homo P3HT (PVTC-PT) Blend Films

**Scheme 1:**
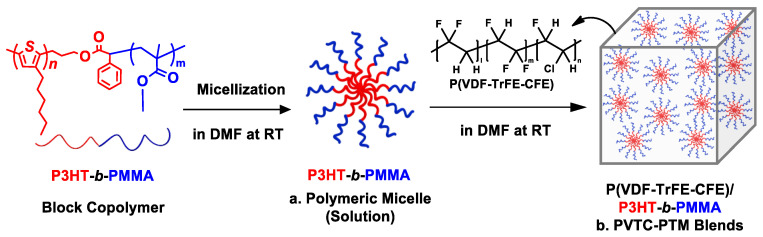
Schematic depiction for P(VDF-TrFE-CFE)/P3HT-*b*-PMMA (PVTC-PTM) blends with a compliant self-assembly process.

The fabrication process for the PVTC-PTM blends is depicted in Scheme 1. The PVTC-PTM blends were prepared by a conventional solution blending method using DMF as a solvent. The P(VDF-TrFE-CFE) polymer matrix (1.0 g) was dissolved in DMF (9 mL) by stirring for 2 h. The prepared P(VDF-TrFE-CFE) solution was mixing with the P3HT-*b*-PMMA block copolymer filler solution, which was prepared by dissolving in the DMF solution for 24 h at room temperature yielding various weight percentages between P(VDF-TrFE-CFE) and P3HT-*b*-PMMA (i.e., 1.0, 1.5, and 2.5 wt%). The blend solutions were dropped onto the glass casting support after filtration using a syringe filter (0.2 μm pore size), and then the casted solutions were dried slowly at 25 °C. Thereafter, the exfoliated blend films were mechanically drawn up to 300% by a uniaxial stretching method and then annealed at 80 °C for 12 h. The PVTC-PT blend film was also prepared using the same procedure for PVTC-PTM blend films.

### 2.3. Electromechanical Measurements

On the basis of our report, the electromechanical transverse actuation strain (S*_t_*) was measured with a laser displacement sensor (Keyence LK-G80, Osaka, Japan). The acquired P(VDF-TrFE-CFE)/P3HT-*b*-PMMA (PVTC-PTM) blend films were positioned by a hand-made displacement sensing system, in which both end-side of the film was softly held by the clamp and the cantilever. The displacement of the film by the electric field was detected by the laser sensor at the cantilever side (0.1 μm sensitivity). The polymer film was under a slight tension for minimized sensing errors from the external force. When the voltage was applied to the electrodes on both sides of the films, the applied films experienced electromechanical deformation, resulting in the change of the cantilever position. The electrochemical measurements were performed by sensing the displacement of the cantilever position. The strain measurements were performed under AC 0.2 Hz electric signal. The electric voltage was applied to the film through both sides of electrodes using a function generator (Agilent 33250A, Santa Clara, CA, USA), and it was amplified with a factor of 1000 through a high voltage lock-in amplifier (Trek 10110B, Lockport, NY, USA). The electromechanical transverse actuation strain (S*_t_*) was defined as the change of length (∆*L*) of the films divided by an initial length of the films (*L*_0_), $S_{t}\left(\%\right)=\frac{\Delta L}{L_{0}}\;\times 100\;\left(\%\right)$. The dimension of the sample films for actuation is 15 mm × 15 mm, and the average thickness was 50 μm. The gold (Au) electrodes on both surfaces of the films were applied by sputtered, and the dimension of the electrodes is 10 mm × 10 mm. Three points were measured, and then the average value was used (the error range was almost less than 2%).

### 2.4. Characterizations

Synchrotron wide-angle X-ray scattering (WAXS) measurements were performed at the 4C2 SAXS beamline of the Pohang Light Source (Pohang, Korea). In situ WAXS data were obtained via data accumulation of 10 s under a predetermined external electric field. An incident X-ray beam was exposed perpendicular to the film surface. Optical microscopy (OM) images were measured by LEICA DM 2500 P (Wetzlar, Germany). Transmission electron microscopy (TEM) examinations were performed using a Tecnai G2 Spirit (FEI Company, Hillsboro, OR, USA) at 120 kV at the Korea Basic Science Institute (Seoul, Korea). Differential scanning calorimetry (DSC) was measured at 10 °C min^−1^ heating rate using Du Pont DSC 2950 (TA Instruments, New Castle, DE, USA). The dielectric constant (K) and dielectric loss (tan *δ*) of the sample films were measured using an Impedance Analyzer (HP1492A, Palo Alto, CA, USA) with a dielectric test fixture (Agilent 151451, Santa Clara, CA, USA). Tensile modulus was determined using a universal testing machine (UTM H5KT, Horsham, PA, USA) with ASTM D-882 standard method with a cross header speed of 10 mm min^−1^. The film thickness was measured with a micrometer (Heidenhain, MT2501, Traunreut, Germany). The sample dimensions for the mechanical tests were 30 mm × 5 mm (length × width).

## 3. Results and Discussion

### 3.1. Fabrication of PVTC-PTM Blend Films

**Figure 1:**
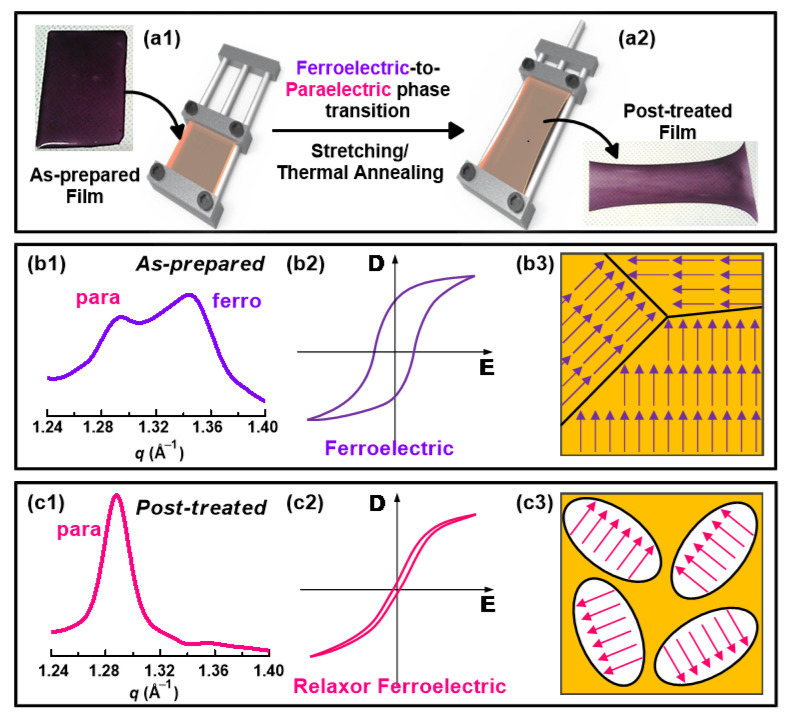
(**a**) Schematic depiction of stretching and thermal annealing process for the actuation film preparation (including photographs for as-prepared and stretched films). The WAXS pattern at the (200, 110) plane, hysteresis loop (D is the electric displacement, E is the electric field), and the illustrations for the crystalline domain structures of PVTC-PTM (**b**) before and (**c**) after the stretching and thermal annealing process (post-treatment).

The P(VDF-TrFE-CFE)/ P3HT-*b*-PMMA (PVTC-PTM) blend films were fabricated by a conventional solution casting method, resulting in about 50 μm in the film thickness (Figure 1a1). First, the P3HT-*b*-PMMA (PTM) block copolymer (*M*_n_: 24,000 g mol^−1^, *M*_w_/*M*_n_: 1.25, P3HT/PMMA: 25/75 wt%) was synthesized on the basis of our previous reports to use for the conducting fillers [30,31]. The acquired PTM solution was applied to the prepared P(VDF-TrFE-CFE) (PVTC) polymer matrix solution in DMF to obtain the homogeneous blend solution. We speculated that the micro-phase separated P3HT-*b*-PMMA could result in a core-shell structure, as shown in Scheme 1a, in which the core and shell layers were constituted by P3HT and PMMA segments, respectively, based on Kim’s report [33,34]. Kim et al. reported that varying the fraction of PMMA in the PTM block copolymers showed microphase separation with different structures, including lamellae, cylinders, and spheres, specifically forming the sphere-like structure in the particular condition of 22 wt% of P3HT with ca. 20 kDa molecular weight of the PTM, yielding the P3HT-PMMA core-shell morphology in the bulk state [33,34]. Besides, the shell layer of PMMA in the structured PTM can provide an excellent opportunity to enhance the compatibility between PTM and PVTC because the hydrogen groups in PVDF-based polymers have been known as enabling a form of strong hydrogen bonds with acryl groups in PMMA, which can help lead to the compatible polymer blends [21,35,36,37]. Their compatible property also addresses preserving the sphere-like micelle structure of the PTM even in the PVTC matrix because of the stabilization of the PMMA shell phase by the encapsulation with the PVTC phase. Those are importantly motivated to design the PVTC-PTM blends, formed by the homogeneous dispersion of PTM in the PVTC matrix (Scheme 1b). The PVTC-PTM blends were prepared with varying PTM content from 1 wt% to 2 wt%. The sample names of PVTC-PTM blends with different PTM content are denoted by putting PTM contents on the end of PVTC-PTM; for example, 1.5 wt% of PTM in the PVTC-PTM is indicated as “PVTC-PTM1.5” hereafter. For comparison, the P(VDF-TrFE-CFE)/P3HT (PVTC-PT) blend film was also prepared using the same method for PVTC-PTM.

The acquired PVTC-PTM films by taking off from the glass support were stretched out up to 300% in a uniaxial way (Figure 1a). The actual film states for as-prepared and post-treated films are displayed in Figure 1a. The stretched film also experienced the thermal annealing process at the same time, resulting in immobilization of the stretched film (Figure 1(a2)). It is a crucial step to imbue the films with crystalline domain relaxation along with the ferroelectric-*to*-paraelectric phase transition. Those are known properties to enable the soft actuator to have the fast polarization response and high-performance electromechanical strain [2,3]. 

The XRD patterns of acquired PVCT-PTM films before and after the stretching and thermal annealing process (post-treatment) can be expected to show significantly different crystalline phase fractions for ferroelectric and paraelectric forces (Figure 1(b1,c1)). The (200, 110) reflections in the pristine PVCT-PTM led to two representative peaks that corresponded to the paraelectric and ferroelectric phases [15,38]. After the stretching process, the paraelectric phase was predominantly grown. In contrast, the ferroelectric phase was almost degraded. The crystalline phase changes can offer unique polarization behavior under turning the electric field on or off. For instance, if the paraelectric phase raised sharply, the permanent dipoles in the PVTC-PTM more rapidly polarized under the electric field than those with enriched ferroelectric phase because of the loss of the ferroelectric phase, as shown in Figure 1(b2,c2). The ferroelectric domain can give rise to the hysteresis in the polarization under the electric field because of the cooperative coupling among ferroelectric domains. Thereby, the PVTC-PTM may experience a quick return to zero polarization upon removing the applied electric field, resulting in a fast mechanical response of the bulk film under the electric field change (Figure 1(c2)) [39,40,41]. 

### 3.2. Compatibility and Crystalline Properties of PVTC-PTM Blends

**Figure 2:**
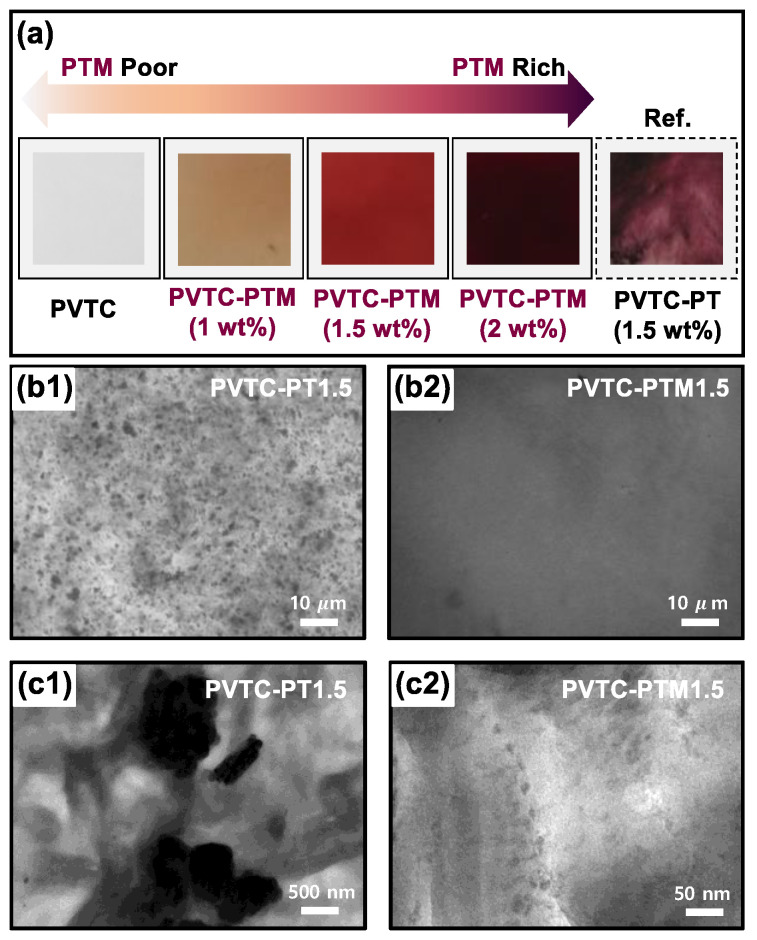
(**a**) Photographs of pristine PVTC, PVTC-PTM1, PVTC-PTM1.5, PVTC-PTM2, and PVTC-PT films. OM images of (**b1**) PVTC-PT1.5 and (**b2**) PVTC-PTM1.5. TEM images of (**c1**) PVTC-PT1.5 and (**c2**) PVTC-PTM1.5.

Figure 2a shows photographs for the resulting films of PVTC, PVTC-PTM1, PVTC-PTM1.5, PVTC-PTM2, and PVTC-PT1.5. The PVTC-PTM blend films exhibit excellent uniformity in color and getting darker in their color with increasing the PTM loading content. That is indicative that the micellized PTM fillers were macroscopically well-dispersed in the PVTC matrix. In contrast, the PVTC-PT1.5 film exhibited a mottled surface in color, which can be derived from the macro-phase separation of PT fillers in the PVTC matrix by their incompatible properties. The OM images of PVTC-PTM1.5 and PVTC-PT1.5 evidenced the PMMA effects on compatibility between PVTC and PTM. The OM image of PVTC-PT1.5 shows large dark spots which are originated from macro-separated P3HT (Figure 2(b1)). On the other hand, the PVTC-PTM1.5 OM image shows relatively uniform contrast (Figure 2(b2)). TEM analysis was also performed to confirm the more microscopic interactive relationship between the PVTC matrix and fillers (Figure 1c). The TEM images of PVTC-PTM1.5 and PVTC-PT1.5 displayed substantially different sizes in the phase-separated fillers (averaged: ca. 10 nm vs. ca. 1 μm, respectively). That also well-corresponded with the results of other microscopic analysis in Figure 2. The compatibility of the conducting fillers in the electrostrictive matrix is an important parameter to enable the uniform local field distribution [21]. Our previous report about the compatibility in the P(VDF-TrFE)/CNT composites importantly discussed the significant effect of the uniform dispersion of the conducting fillers on the electromechanical properties [21].

**Figure 3:**
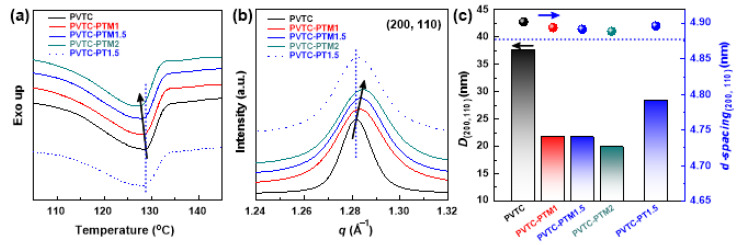
(**a**) DSC curves and (**b**) WAXS patterns of PVTC, PVTC-PTM1, PVTC-PTM1.5, PVTC-PTM2, and PVTC-PT1.5 under N_2_. (**c**) Crystalline domain size and d-spacing plots of PVTC, PVTC-PTM1, PVTC-PTM1.5, PVTC-PTM2, and PVTC-PT1.5 based on the (200, 110) crystalline plane.

The compatibility of PTM with the PVTC matrix was examined by DSC. The DSC curves were acquired by a second run process with a heating rate of 10 °C min^−1^. The obtained endothermic peak in PVTC was observed at 127.8 °C, which corresponded to the melting point (*T*_m_) of the PVTC. The blending of the PVTC with the PTM led to decreased *T*_m_ from 127.5 to 128.9 °C with an increase of the PTM loading content from 1 to 2 wt% (Figure 3a). Our previous reports importantly discussed the behavior of the *T*_m_ decrease in PVDF-based polymers by the compatible blend, which originated from the reduction of the crystal size in the lamellar crystalline structure of PVTC [21,35,37]. In contrast, PVTC-PT1.5 exhibited almost similar *T*_m_ (128.7 °C) to that of PVTC. It indicates the lesser effectiveness of PT for the crystalline structure alteration of PVTC than that of PTM.

The crystalline properties significantly affect the electromechanical properties of electrostrictive polymers [38]. In addition, the crystal grain size is also an essential parameter in the polarization behavior as a function of the electric field. The crystalline properties of the PVTC and its blends were examined by WAXS, as shown in Figure 3b. The WAXS pattern of the PVTC showed a strong and sharp peak at 1.281 Å^−1^, which corresponded to the (200, 110) crystalline plane. After the blending of the PVTC with the PTM, the peak was getting broader and shifted to the higher *q* (scattering vector) value with increasing loading content of the PTM. Those facts also prove that the PTM influenced the crystalline structure of the PVTC by the inter-chain interaction between them. Although PVTC-PT1.5 exhibited a broader peak than that of PVTC, the peak position was not much shifted (*q* = 1.2825 Å^−1^) relative to that of the PVTC; those outcomes are almost identical to the DSC results (Figure 3a,b).

On the basis of WAXS patterns, the average crystalline domain size was calculated by the Scherrer formula: *D* = $K\lambda /\beta_{hkl}cos\theta$, where *D* is the average crystalline size, *K* is a shape factor (1), *λ* is the wavelength of CuKα radiation (1.54 Å) and $\beta_{hkl}$ is full width of half maximum (FWHM). The crystalline domain size of PVTC was calculated to be 38.52 nm and substantially reduced to 21.72 nm in the presence of 1 wt% of the PTM, and it sequentially decreased to 19.87 through the addition of 2 wt% of the PTM to the PVTC matrix (Figure 3c and Table 1). We anticipated that the smaller crystal grain size could give rise to the faster polarization response by the applied electric field and the denser crystal grain packing in the electrostrictive polymers in comparison to those of the polymers with the larger sized crystal grain [38,42]. Hence, the design of the PVTC-PTM probably can lead to enhanced electromechanical performance in polarization hysteresis and higher displacement under the electric field, compared to that of the PVTC. In contrast, the PVTC-PT1.5 showed a relatively larger crystal grain size (28.28 nm) relative to that of PVTC-PTM1.5 (21.28 nm), indicating that the design of the PVTC-PT may be less significant to electromechanical property enhancement than that of the PVTC-PTM.

The *d*-spacing of the PVTC and its blends was calculated to be around 4.9 nm, which means that they are almost identical (Figure 3c and Table 1). However, the marginal decrease trend in the *d*-spacing was observed by increasing the PTM loading content (Figure 3c). In addition, the *d*-spacing of the PVTC-PT1.5 (4.897 nm) was relatively more extensive than that of PVTC-PTM1.5 (4.292 nm), albeit they are not substantially different (Figure 2c). With regard to that, Zhang et al. reported that it is because of the lower nonpolar conformation [12,43]. On the basis of the melting peak in DSC, the crystallinity (*X*_c_) of PVTC and its blends was calculated [21,37]. The calculated crystallinity of PVTC was 15.5%. It smoothly increased to 22.2% with increased PTM loading content up to 2 wt%; we expected a more substantial electrostriction effect by the higher polar phase content than the PVTC (Table 1). Meanwhile, the PVTC-PT1.5 (16.9%) exhibited relatively lower crystallinity than that of PVTC-PTM1.5 (19.1%). We can anticipate that the enhanced polar crystallinity of PVTC by the addition of PTM may lead to the improved electromechanical strain of PVTC-PTM relative to PVTC and PVTC-PT because of the enhanced electrostriction force.

### 3.3. Electromechanical Properties of PVTC-PTM Blends

**Figure 4:**
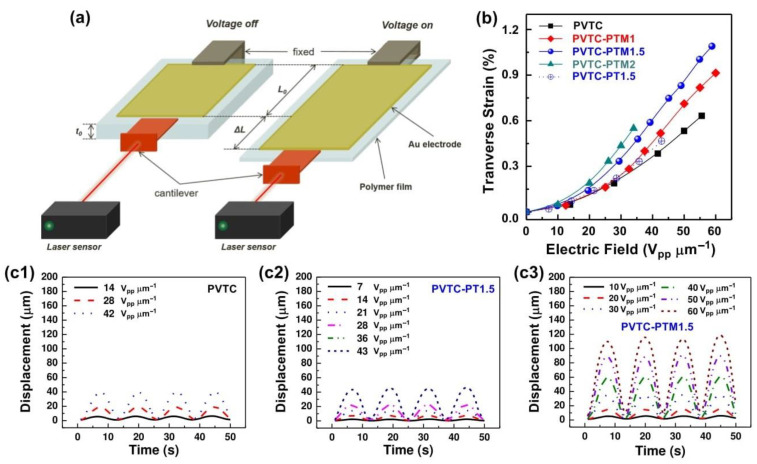
(**a**) Illustration of the transverse strain measurement system. (**b**) Transverse strains of PVTC, PVTC-PTM1, PVTC-PTM1.5, PVTC-PTM2, and PVTC-PT1.5 as functions of the electric field. Displacement curves of (**c1**) PVTC, (**c2**) PVTC-PT1.5, and (**c3**) PVTC-PTM1.5 as functions of the applied electric field at 0.2 Hz of frequency.

On the basis of the studies for the PVTC-PTM crystalline properties, we expected that the PVTC-PTM blends would have the better electromechanical performance in comparison to the PVTC and PVTC-PT because of the smaller crystal grain size and the higher polar crystalline properties (Figure 3c and Table 1) [15,38]. To confirm our hypothesis, the electromechanical performances of the PVTC and its blends were evaluated under the different electric fields. The electromechanical operation system was set up as depicted in Figure 4a, where the sample was loaded between the holder and the cantilever. The displacement was measured using a laser sensor under the control of the voltage on and off (0.2 Hz AC electric signals). Having acquired displacement changes, the transverse strain (*S*) was calculated by the following equation: *S* (%) = (∆*L*/*L_0_*) × 100 (%), where ∆*L* is the displacement of the films by the electric field and *L*_0_ is the transverse length of the original film.

Figure 4b shows transverse strain changes of the PVTC and its blends as functions of the electric field. The increase of the PTM loading content in the PVTC-PTM led to increased transverse strain in the same electric field. For instance, the transverse strain of PVTC was 0.22% at 30 V_pp_ μm^−1^, followed by PVTC-PTM1 (0.24%), PVTC-PTM1.5 (0.35%), and PVTC-PTM2 (0.44%) (Figure 4b and Table 2). For comparison, PVTC-PT1.5 was also evaluated for its electromechanical strain, resulting in 0.25% at 30 V_pp_ μm^−1^. Although the result by PVTC-PT1.5 is slightly higher than that of pristine PVTC (0.22% at 30 V_pp_ μm^−1^), it is substantially lower than that of PVTC-PTM1.5 (0.35% at 30 V_pp_ μm^−1^). The actual displacement signals of the PVTC, PVTC-PT1.5, and PVTC-PTM1.5 films as functions of the electrical field are displayed in Figure 4c. All samples showed uniform displacement trace according to the electric field’s difference, indicating that the displacement signals are reliable [21]. In particular, we can confirm that PVTC-PTM1.5 showed the highest displacement in a similar electric field relative to those of PVTC and PVTC-PT1.5 (Figure 4c).

**Figure 5:**
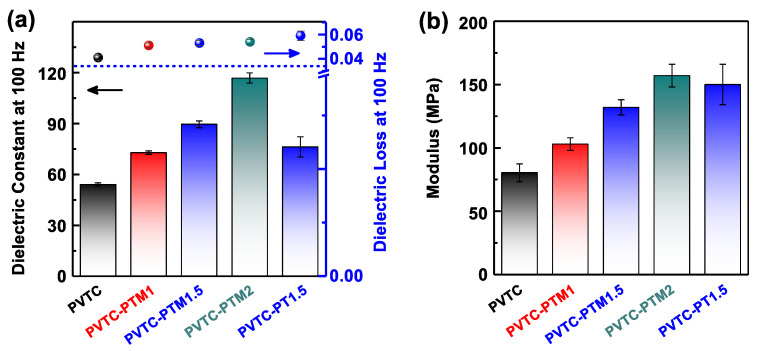
(**a**) Dielectric properties (the real part of the dielectric constant and dielectric loss) and (**b**) moduli of PVTC, PVTC-PTM1, PVTC-PTM1.5, PVTC-PTM2, and PVTC-PT1.5 at room temperature.

The relative evaluation was performed to understand the achievement of our current study. In Table 2, the traditional electroactive polymers, including PVDF, silicone, and acrylic polymers, were employed for the relative evaluation [20,44,45,46]. For a precise comparison of our material state, the transverse strain value at 30 V_pp_ μm^−1^ was added to Table 2. The PVTC-PTM blends showed a relatively higher transverse strain value than other reference materials, including PVDF, silicone, and acrylic polymers. In particular, the PDMS/CNT (0.01 wt%) composite showed a comparable value, ca. 0.4%. However, the inorganic conducting filler’s (CNT) addition led to a significantly low electrical breakdown strength (30 V_pp_ μm^−1^), resulting in a low operation electric field range [20]. It can be speculated that the form of the conducting path in the composite gave rise to the prone electrical connection between electrodes. The results were importantly motivated by designing the PVTC-PTM blends mainly using organic fillers, not inorganic fillers. However, we inevitably observed similar behaviors in the PVTC-PTM blend by adding a large amount of the PTM filler (2 wt%), exhibiting the relatively lower electrical breakdown strength at ca. 35 V_pp_ μm^−1^ (PVTC-PTM2). In addition, the PVTC-PT1.5 also showed a relatively lower electrical breakdown strength (42 V_pp_ μm^−1^) than that of the PVTC and the PVTC-PTM1.5 (Figure 4b). That was probably due to the insufficient dispersion properties of fillers in the PVTC matrix, which can be discussed in microscopic studies (Figure 2) [47]. The electrical breakdown strength is correlated with dielectric properties, particularly dielectric loss. The PVTC-PTC1.5 showed a relatively higher dielectric loss value (0.059) than that of the PVTC-PTM1.5 (0.053), albeit they are marginally different (Figure 5a). The materials with high dielectric loss are commonly understood to also offer low electrical breakdown strength. As such, the design of the PVTC-PTM with 1.5 wt% of the PTM filler can be said to be the optimized blend system in the current study.

The principle mechanisms leading to electromechanical properties in electroactive polymers are known as electrostatic and electrostrictive forces; specifically, (1) electrostatic force (*S* = 0.5*Kε_0_E*^2^ (1 + 2*σ*)/*Y*, where *K* is the dielectric constant, *ε*_0_ is the vacuum dielectric permittivity (8.85 × 10^−12^ F m^−1^), *E* is the applied electric field, *σ* is the Poisson’s ratio, and *Y* is the elastic modulus); (2) electrostrictive force (*S* = *ME*^2^, where *M* is the field related electrostrictive coefficient, which is proportional to the square of the dielectric constant (*K*)) [21]. Based on the mechanisms for electromechanical strain, the key parameters can be defined as increasing electrostatic force and electrostrictive force, which are influenced by modulus and dielectric constant, respectively [21]. To confirm this, we measured the moduli and dielectric properties of PVTC, PVTC-PTM1, PVTC-PTM1.5, PVTC-PTM2, and PVTC-PT1.5, as shown in Figure 5. The PVTC matrix with an increase of the PTM filler loading content (up to 2 wt%) led to the rise of dielectric constant from 54.1 to 116.8 at 100 Hz and room temperature (Figure 5a). It affected the dramatic increase in electrostrictive force because it is proportional to the square of the dielectric constant. However, the PTM’s addition to the PVTC for PVTC-PTM blends gave rise to the slight increase in the modulus value from 0.08 GPa (PVTC) to 0.157 GPa (PVTC-PTM2), resulting in a decrease of electrostatic force; there is an inversely proportional relation between them (Figure 5b). The enhanced electromechanical strain of the PVTC through the design of the PVTC-PTM blends can be addressed by the improved electrostrictive force, albeit with a little sacrifice in the electrostatic force. Moreover, the relatively lower electromechanical performance of the PVTC-PT1.5 than that of PVTC-PTM1.5 can also be addressed in the same regard because it showed a somewhat higher modulus and lower dielectric constant than those of PVTC-PTM1.5.

The field-related electrostrictive coefficient (*M*) was calculated for PVTC and its blends, causing the PVTC-PTM2 to reveal the superior value (4.9 × 10^−4^ μm^2^ V^−2^). In addition, the *M* of the PVTC-PTM1.5 (3.9 × 10^−4^ μm^2^ V^−2^) was more significant than that of PVTC-PT1.5 (2.8 × 10^−4^ μm^2^ V^−2^) (Table 2). The evaluation of *M* in PVTC and its blends is almost consistent with the electromechanical results. It also confirms the electrostrictive dominant operation mechanism in the PVTC-PTM blends. The strained elastic energy density for PVTC was calculated to be 0.19, based on the equation reported by Zhang et al. (*U*_s_ = 0.5*YS*^2^). The *U*_s_ value of the PVTC increased by the addition of PTM and PT fillers (Table 2). In addition, the *U*_s_ value indicates the stored elastic energy density when a polymer is strained [7]. As such, we speculate that the addition of the PTM and PT fillers into the PVTC can provide a more massive transverse strain than that of the neat PVTC. The *U*_s_’ calculation can be a theoretical clue to confirm why the PVTC-PTM showed the enhanced electromechanical performance relative to the PVTC and the PVTC-PT blend. As such, the PTM is the more effective filler than that of the PT for upgrading the transverse strain of the PVTC. Zhang et al. discussed the conducting filler effects on electrostrictive polymers, resulting in the importance of uniform local field distribution, which is offered by uniform dispersion of the conducting fillers in electrostrictive polymers [44]. In addition, they also importantly mentioned the easy displacement of the electrons under the electric field, which is affected by the enhanced electron delocalization within the conducting fillers [7].

### 3.4. Electromechanical Strain Mechanism Study

**Figure 6:**
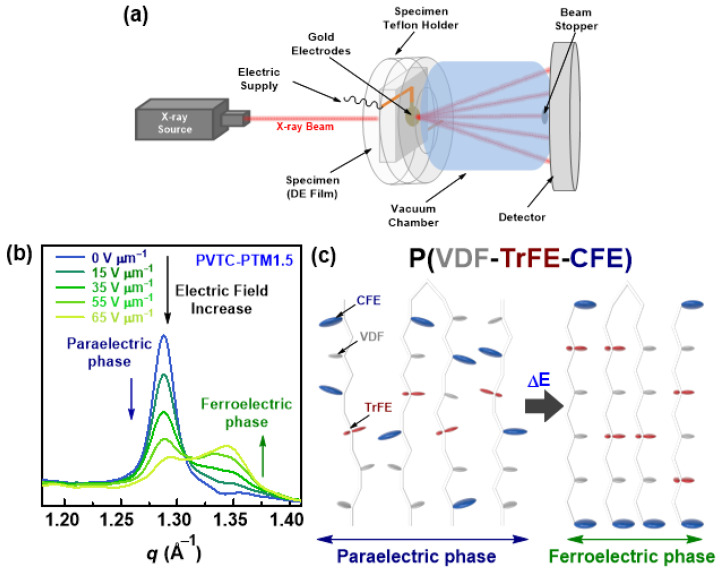
(**a**) Illustration for WAXS measurement as a function of the applied electric field at room temperature. (**b**) WAXS patterns of PVTC-PTM1.5 corresponding to the applied electric field. (**c**) Schematic representations of the laterally expanded P(VDF-TrFE-CFE) crystalline domain at the paraelectric phase and ferroelectric phase (para-*to*-ferro transition by an electric field).

The electromechanical properties of the PVCT-PTM blends were evaluated on the basis of the parameter differences regarding other reference materials. In addition, we significantly discussed the electromechanical strain mechanism of PVCT-PTM blends with abundant evidence to reveal what parameters specifically affected their electromechanical operation mechanism. The mechanism was seen to be dominantly affected by the electrostrictive forces from the enhanced dielectric properties of PVTC by adding the PTM conducting filler. Those can be acceptable in the common understanding from reported PVDF-based electromechanical studies [49,50,51]. Moreover, the electron delocalization in the electrostrictive polymers can help the fast electron displacement within the polar groups in the polymer chains [7]. It corresponded to the quick response to the polar group alignment in PVTC polymer chains [7]. The WAXS studies with the PVTC-PTM blend can also give a significant clue to understanding the electromechanical strain in the bulk film. The crystalline phase transition from paraelectric to ferroelectric can give rise to the alteration of the micro-crystalline domain volume by their unique alignment of polymer chains, which well-linked to the displacement of the bulk state film [14,38]. To confirm our suggestion in the relationship between the crystalline phase change and electro-mechanical strain, we set up the WAXS measurement system under the electric field. The Teflon insulator enclosed the PVTC-PTM1.5 sample; it was located at the center of the C-ray beamline along with the electric supply connection to the electrodes on both sides of the film (Figure 6a). WAXS patterns of the PVTC-PTM1.5 were acquired under a particular electric field with a minute retention time. The resulting WAXS patterns are displayed in Figure 6b. The WAXS pattern of the PVTC-PTM1.5 without the electric field showed the paraelectric dominant crystalline phase. The ferroelectric phase in the PVTC-PTM1.5 was smoothly grown up by an increase of the electric field, and it then showed a decrease of the paraelectric phase. The crystalline phase transition from paraelectric to ferroelectric was observed at the electric field of 55 V μm^−1^ (Figure 6b). Figure 6c introduces what changes in the crystalline domain of the PVTC by the para-to-ferroelectric phase transition. The polar group in the crystalline domain of the PVTC film experienced good alignment along the same direction under the electric field. The alignment of the polar groups in polymer chains eventually affords the rearrangement of the bulk film volume. It affects the transverse strain by increasing the length of the PVTC-PTM1.5 bulk film. We believe that the WAXS study provides essential information to address the electromechanical strain mechanism of the PVTC-PTM blend.

## 4. Conclusions

The PVTC-PTM blends were fabricated by the conventional solution casting method, resulting in the excellent compatibility between PVTC and PTM by forming a hydrogen bond between them. That was directly proven by microscopic studies and supported by DSC thermal analysis, through showing a decrease of the PVTC melting point with increasing the PTM loading content. In addition, we found that the addition of the PTM into the PVTC led to an increase of the polar crystallinity and a reduction in the crystal grain size compared to those of the PVTC. Moreover, the PVTC-PTM blends exhibited a substantially high dielectric constant 116.8 at 100 Hz, which is a 2-fold increase relative to PVTC. It is indicative that the PVTC-PTM blends can be more affected by electrostrictive force than PVTC. Indeed, the PVTC-PTM1.5 showed a much larger transverse strain (0.44% at 30 V_pp_ μm^−1^) than that of PVTC (0.22% at 30 V_pp_ μm^−1^), although the modulus of the PVTC-PTM1.5 (0.132 GPa) was larger than that of PVTC (0.08 GPa). The enhanced electromechanical property of the PVTC-PTM blend originates from the enhanced uniform local field distribution through the homogenously dispersed PTM conducting fillers in the PVTC matrix. It affects the rearrangement of polar groups in the polymer chains, which is linked to the bulk film volume changes. The mechanism for the electromechanical strain of the PVTC-PTM blends was confirmed by the WAXS study, exhibiting that the applied field to the PVTC-PTM film gave rise to the crystalline phase transition from paraelectric to ferroelectric. In other words, the crystalline phase transition affected the volume change of the bulk film toward a transverse strain way by the rearrangement of the polar groups in the polymer chains.

## Figures and Tables

**Table 1:** Structural parameters of PVTC and its blends.

Entry	2θ	*d* (nm)	hkl	FWHM (rad)	*D*^a^ (nm)	*X*_c_^b^ (%)
PVTC	18.08	4.902	(200, 110)	0.0043	38.52	15.5
PVTC-PTM1	18.11	4.894	(200, 110)	0.0076	21.72	17.4
PVTC-PTM1.5	18.12	4.892	(200, 110)	0.0076	21.68	19.1
PVTC-PTM2	18.13	4.889	(200, 110)	0.0083	19.87	22.2
PVTC-PT1.5	18.10	4.897	(200, 110)	0.0058	28.28	16.9

^a^*D* was deduced from Scherrer formula; ^b^ *X*_c_ = Δ*H*_m_/[(1 − *W_0_*) Δ*H*_0_] × 100 (%), where *W_0_* is the weight fraction of the filler in the blends and Δ*H*_0_ is the heat of fusion for 100% crystalline PVDF (104.7 J g^−1^). The error range is less than 5%.

**Table 2:** Profiles for deducing the energy conversion rate.

	Entry	Filler Content (wt%)	*K* at 100 Hz	*Y* (GPa)	Transverse Strain ^a^ (%)	*M*^b^ (μm^2^ V^−2^, ×10^−4^)	*U*_S_ (kJ cm^−3^)	Ref.
PVDF Polymers	PVTC	0.0	54.1	0.08	0.22	2.4	0.19	This Work
	PVTC-PTM1	1.0	72.8	0.103	0.24	2.7	0.30	This Work
	PVTC-PTM1.5	1.5	89.6	0.132	0.35	3.9	0.81	This Work
	PVTC-PTM2	2.0	116.8	0.157	0.44	4.9	1.52	This Work
	PVTC-PT1.5	1.5	76.3	0.150	0.25	2.8	0.47	This Work
	P(VDF-TrFE) (68/32)	-	14.0	0.4	0.20	-	-	[44]
Silicone Rubbers	PDMS	-	3.3 ^c^	0.0002	0.25	-	-	[48]
	PDMS/CNT	0.01	3.4 ^c^	0.00024	0.40	-	-	[20]
Acrylic Polymers	PMDM	-	3.4	0.00017	0.035	-	-	[45]
	PTMDMT	-	3.8	0.00021	0.11	-	-	[46]
	PTDT	-	3.9	0.00052	0.07	-	-	[46]

^a^ Electromechanical strain at 30 V_pp_ μm^−1^. ^b^ The *M*, field related electrostrictive coefficient, the value of examined samples was calculated by using the equation of *S* = *ME*^2^. ^c^ Dielectric constant at 100 kHz.
